# Reliable Finite Frequency Filter Design for Networked Control Systems with Sensor Faults

**DOI:** 10.3390/s120607975

**Published:** 2012-06-08

**Authors:** He-Hua Ju, Yue Long, Heng Wang

**Affiliations:** 1 College of Electronic Information and Control Engineering, Beijing University of Technology, Beijing 100124, China; E-Mail: juhehua@bjut.edu.cn; 2 College of Information Science and Engineering, Northeastern University, Shenyang, Liaoning 110004, China; E-Mail: lemonshell@yahoo.cn

**Keywords:** reliable filtering, networked control system, data missing, quantization, sensor faults

## Abstract

This paper is concerned with the reliable finite frequency filter design for networked control systems (NCSs) subject to quantization and data missing. Taking into account quantization, possible data missing and sensor stuck faults, NCSs are modeled in the framework of discrete time-delay switched systems, and the finite frequency *l*_2_ gain is adopted for the filter design of discrete time-delay switched systems, which is converted into a set of linear matrix inequality (LMI) conditions. By the virtues of the derived conditions, a procedure of reliable filter synthesis is presented. Further, the filter gains are characterized in terms of solutions to a convex optimization problem which can be solved by using the semi-definite programme method. Finally, an example is given to illustrate the effectiveness of the proposed method.

## 1. Introduction

In recent years, there has been a growing interest in networked control systems (NCSs), which is a class of systems in which sensors, controllers and plants are connected over the network media [1–4]. Due to their advantages such as easy installation, low cost and high utilization, the NCSs have widely applications in many application areas, such as manufacturing plants, automobiles and remote process, *etc.* However, these systems require novel control design to account for the presence of network in the closed loop, such as network-induced delay (see e.g., [5–8]) and packet loss (see e.g., [9,10]). Further, for the NCSs where bandwidth and energy are limited, quantization becomes indispensable. Consequently, there has been a lot of researches concerning this issue, (see e.g., [11,12]).

On the other hand, filtering problem has been playing an important role in control engineering and signal processing that has attracted constant research attention, (see e.g., [13–17] and references therein). However, it is quite common in practice that measurement outputs of a dynamic system contain incomplete observations because of the temporal sensor faults, (see e.g., [18–21] and references therein). Therefore, it is natural that the reliable filtering problem in presence of possible sensor faults has recently obtained much attention and there have been many results investigating this important issue. For example, reliable filtering problems have been thoroughly investigated in [22–24] for linear systems. As for nonlinear systems, reliable filtering problems with sensor faults have also attracted many research interests [25–27].

It should be noted that disturbances considered in those papers are all considered in full frequency domain. However, practical industry systems often employ large, complex, or lightweight structures, which include finite frequency fundamental vibration modes. Thus, it is more reasonable to design reliable filters in finite frequency domain. However, to the best of the authors' knowledge, reliable filtering problems for NCSs subject to packet loss and quantization have not been fully investigated, especially in finite frequency domain where faults occur frequently. This motivates the investigation of this work.

In response to the above discussions, in this paper, the reliable finite frequency filtering problem for NCSs subject to packet loss and quantization is investigated in finite frequency domain against sensor stuck faults. Specifically, with consideration of quantization, possible packet losses and possible sensor stuck faults, NCSs are modeled in a framework of discrete time-delay switched system. Then, the definition of finite frequency *l*_2_ gain is given and an analysis condition to capture such a performance for discrete time-delay switched system is derived. With the aid of the derived conditions, a reliable filter is designed and the conclusions are presented in terms of linear matrix inequalities (LMIs). Finally, an example is given to illustrate the effectiveness of the proposed method.

The reminder of the paper is organized as follows. The problem of system modeling for NCSs with packet losses and quantization is presented in Section 2. Section 3 provides sufficient conditions for the design of reliable filters. In Section 4, an example is given to illustrate the effectiveness of the proposed method. Finally, some conclusions are presented in Section 5.

### Notations

Throughout the paper, the superscript *T* and −1 stand for, respectively, the transposition and the inverse of a matrix; *M* > 0 means that *M* is real symmetric and positive definite; *I* represents the identity matrix with compatible dimension; ‖·‖ denotes the Euclidean norm; ℙ is the probability measure; ℝ(·) denotes the expectation operator; *l*_2_ denotes the Hilbert space of square integrable functions. In block symmetric matrices or long matrix expressions, we use * to represent a term that is induced by symmetry; The sum of a square matrix *A* and its transposition *A^T^* is denoted by *He*(*A*):= *A* + *A^T^*.

## 2. System Model and Problem Formulation

**Figure 1.**
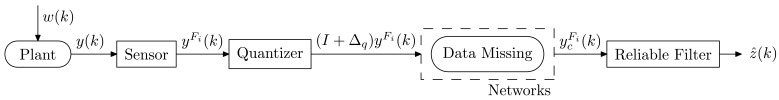
Structure of the Networked Control Systems.

The NCS under consideration is setup in Figure 1, where the discrete-time plant is of the form:

(1) $$\begin{array}{cc} x(k+1) & =Ax(k)+Bw(k)\\ y(k) & =Cx(k)\\ z(k) & =Ex(k) \end{array}$$

where *x*(*k*) ∈ **R***^n^* is the state, *y*(*k*) ∈ **R***^m^* is the measured output, *z*(*k*) ∈ **R***^p^* is the controlled output and *w*(*k*) ∈ **R***^d^* is the exogenous disturbance which is assumed to belong to *l*_2_[0, ∞). *A, B, C* and *E* are known real constant matrices with appropriate dimensions.

In this paper, we make the following assumption:

### Assumption 1

System (1) is stable.

### Remark 2

Assumption 1 is required to get stable dynamics of the filter system. If this assumption is not satisfied, a stabilizing output feedback controller is required.

When the sensors in the NCSs experience faults, we consider the following sensor stuck fault model similar to [28],

(2) $$\begin{array}{ll} y^{Fi}(k)=F_{i}y(k)+(I-F_{i})y_{si}(k), & i=0,1,2,\ldots ,q \end{array}$$

where *q* is the quantity of the possible fault modes and

(3) $$y_{si}(k)={\begin{array}{cccc} \left[y_{si1}(k\right) & y_{si2}(k) & \ldots & y_{sim}(k)] \end{array}}^{T}$$

with *y_sij_*(*k*)(*j* = 1, 2,…, *m*) being the low frequency fault of the *k*th sensor. Further, *F_i_* is defined as

(4) $$\begin{array}{c} F_{i}=\text{diag}\;\{F_{i1},F_{i2},\ldots ,F_{im}\}\\ F_{ik}=\begin{array}{cc} 0\;\text{or}\;1\;, & k=1,2,\ldots ,m \end{array} \end{array}$$

It is also assumed that, as shown in Figure 1, the measurement signals will be quantized before transmitting via the networks wherein data missing may occur. The following logarithmic quantizer as proposed in [29] is applied,

(5) $$q(\upsilon )=\{\begin{array}{ll} \rho^{i}\upsilon_{0} & \text{if}\;\frac{1}{1+\delta_{q}}\rho^{i}\upsilon_{0}<\upsilon \leq \frac{1}{1-\delta_{q}}\rho^{i}\upsilon_{0}\\ 0 & \text{if}\;\upsilon =0\\ -q(\upsilon ) & \text{if}\;\upsilon <0 \end{array}$$

where the parameter 0 < *ρ* < 1 is termed as *quantization density* and

(6) $$\delta_{q}=(1-\rho )/(1+\rho )$$

From [29], we can obtain

(7) $$q(\upsilon )=(I+\Delta_{q})\upsilon$$

where Δ*_q_* ∈ [−*δ_q_,δ_q_*] is a suitable model for the logarithmic quantizer *q*(*υ*) with parameter *δ_q_*.

Therefore, the faulty measurements together with quantization and the data transmission in the networks can be described by

(8) $$y_{c}^{Fi}(k)=\alpha (I+\Delta_{q})y^{Fi}(k)+(1-\alpha \;)(I+\Delta_{q}\;)y^{Fi}(k-1)$$

where *α* ∈ **R** is a Bernoulli distributed white sequence with

$$\begin{array}{c} \mathbb{P}(\alpha =1)=𝔼(\alpha )=\bar{\delta }\\ \mathbb{P}(\alpha =0)=1-𝔼(\alpha )=1-\bar{\delta } \end{array}$$

Specifically, if *α* = 1, the quantized signal (*I* + Δ*_q_*)*y^Fi^*(*k*) is successfully transmitted, otherwise the transmission fails, *i.e.*, the phenomenon of *data missing*.

### Remark 3

The description of data transmission (8) was introduced in [30]. It can be seen that the output *y*(*k*) of the system model is (*I* + Δ*_q_*)*y^Fi^*(*k*) with probability *δ̄* at *k-th* sampling time, and the value (*I* + Δ*_q_*)*y^Fi^*(*k* − 1) with probability 1 − *δ̄* Obviously, if the binary stochastic variable α takes the value *0* consecutively at different sample times, the consecutive data missing would occur.

In this paper, the following reliable filter is constructed:

(9) $$\begin{array}{c} \tilde{x}(k+1)=A_{f}\tilde{x}(k)=B_{f}y_{c}^{Fi}(k)\\ \tilde{z}(k)=C_{f}\tilde{x}(k) \end{array}$$

where *A_f_, B_f_* and *C_f_* are filter parameters to be designed.

Denoting *ζ*(*k*) = [*x^T^*(*k*) *xˆ^T^*(*k*)]*^T^* and *e*(*k*) = *z*(*k*) – *z̃*(*k*), then the filtering error system for the *i*th fault mode can be described by the following two subsystems.

*S*_1_: No packet dropout occurs.
  $$\begin{array}{cc} \zeta (k+1) & ={\text{A}}_{1i}\zeta (k)+{\text{A}}_{1di}\zeta (k-1)+{ℬ}_{w}w(k)+{ℬ}_{si}y_{si}(k)\\ e(k) & =\text{C}\zeta (k) \end{array}$$
*S*_2_: Packet dropout occurs.
  $$\begin{array}{cc} \zeta (k+1) & ={\text{A}}_{2i}\zeta (k)+{\text{A}}_{2di}\zeta (k-1)+{ℬ}_{w}w(k)+{ℬ}_{si}y_{si}(k)\\ e(k) & =\text{C}\zeta (k) \end{array}$$
  where
  $$\begin{array}{cc} \left[{\text{A}}_{1i}|{\text{A}}_{1di}|{\text{A}}_{2i}|{\text{A}}_{2di}\right] & =\left[\begin{array}{cc} A & 0\\ B_{f}(I+\Delta_{q})\;F_{i}C & A_{f} \end{array}|\begin{array}{cc} 0 & 0\\ 0 & 0 \end{array}|\begin{array}{cc} A & 0\\ 0 & A_{f} \end{array}|\begin{array}{cc} 0 & 0\\ B_{f}\;(I+\Delta_{q})\;F_{i}C & 0 \end{array}\right]\\ \left[{ℬ}_{w}|{ℬ}_{si}\right] & =\left[\begin{array}{c} B\\ 0 \end{array}|\begin{array}{c} 0\\ B_{f}(I+\Delta_{q}\;)(I-F_{i}) \end{array}\right]\\ \text{C} & =[E-C_{f}] \end{array}$$

Due to packet drop-out, the filtering error system can be seen as combined by subsystem *S*_1_ and *S*_2_, which can be lumped into the following discrete time-delay switched system:

(10) $$\begin{array}{cc} \zeta (k+1) & ={\text{A}}_{\sigma_{k}i}\zeta (k)+{\text{A}}_{\sigma_{k}di}\zeta (k-1)+{ℬ}_{w}w(k)+{ℬ}_{si}y_{si}(k)\\ e(k) & =\text{C}\zeta (k) \end{array}$$

where *σ_k_* is *switching signal* with *σ_k_* ∈ 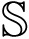 = {1, 2} being a piecewise constant function.

Next, we will discuss how to design the filter parameters *A_f_, B_f_* and *C_f_*. In order to formulate the problem clearly, the following definitions are first given.

### Definition 4. (Asymptotical Stable)

System (10) is said to be asymptotical stable under switching signal σ_k_, if the solution satisfies

(11) $$\underset{k\to 0}{\lim}\left\|\zeta (k)\right\|=0$$

### Definition 5

Ding *et al.* [17] Let *γ* > 0 be a given constant, then the filtering error system (10) is said to have a finite-frequency *l*_2_ gain *γ*, if inequality

(12) $$\sum_{k=0}^{\infty }e{\left(k\right)}^{T}e(k)\leq \gamma^{2}\sum_{k=0}^{\infty }w{\left(k\right)}^{T}w(k)$$

holds for all solutions of Equation (10) with w(*k*) ∈ *l*_2_ such that the following hold

- For the low-frequency range |*θ*| < *ϑ_l_*
  (13) $$\sum_{k=0}^{\infty }(\zeta (k+1)-\zeta (k))\;{\left(\zeta (k+1)-\zeta (k)\right)}^{T}\leq {\left(2\sin\frac{\vartheta_{l}}{2}\right)}^{2}\sum_{k=1}^{\infty }\zeta (k)\zeta {\left(k\right)}^{T}$$
- For the middle-frequency range *ϑ*_1_ ≤ *θ* ≤ *ϑ*_2_
  (14) $$e^{j\vartheta_{w}}\sum_{k=0}^{\infty }(\zeta (k+1)-e^{j\vartheta_{1}}\zeta (k))\;{\left(\zeta (k+1)-e^{-j\vartheta_{2}}\zeta (k)\right)}^{T}\leq 0$$
  where *ϑ_w_* = (*ϑ*_2_ − *ϑ*_1_)/2.
- For the high-frequency range |*θ*| ≥ *ϑ_h_*
  (15) $$\sum_{k=0}^{\infty }(\zeta (k+1)-\zeta (k))\;{\left(\zeta (k+1)-\zeta (k)\right)}^{T}\geq {\left(2\sin\frac{\vartheta_{h}}{2}\right)}^{2}\sum_{k=0}^{\infty }\zeta (k)\;\zeta {\left(k\right)}^{T}$$

Now, the reliable filtering problem to be addressed in this paper can be formulated as follows:

Design a stable reliable filter (9) such that, for the quantization error, possible data missing and sensor faults, the filtering error system (10) is asymptotical stable, and with a prescribed finite-frequency *l*_2_ gain *γ*_1_ from *w*(*k*) to *e*(*k*) by satisfying the following specification

(16) $$\begin{array}{ll} \sum_{k=0}^{\infty }{\left\|e(k)\right\|}_{2}\leq \gamma_{1}^{2}\sum_{k=0}^{\infty }{\left\|w(k)\right\|}_{2}, & \forall \theta_{w} \end{array}$$

and a prescribed low frequency *l*_2_ gain *γ*_2_ from *y_si_*(*k*) to *e*(*k*) by satisfying

(17) $$\begin{array}{cc} \sum_{k=0}^{\infty }{\left\|e(k)\right\|}_{2}\leq \gamma_{2}^{2}\sum_{k=0}^{\infty }{\left\|y_{si}\;(k)\right\|}_{2}, & \forall \left|\theta_{s}\right|\leq \vartheta_{sl} \end{array}$$

where *θ_w_* and *θ_s_* represent the frequency of the disturbance and the stuck faults, respectively.

Before ending this section, the following lemmas will be first given to help us to prove our main results.

### Lemma 6. (Finsler's Lemma)

For *x* ∈ **R***^n^*, 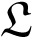 ∈ **R***^n^*^×^*^n^*, 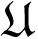 ∈ **R***^n^*^×^*^m^*, let 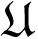
^⊥^ be any matrix such that 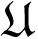
^⊥^ 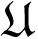 = 0. The following statements are equivalent:

- *x^T^* 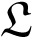
*x* < 0, ∀ 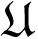
*^T^x* = 0, *x* ≠ 0,

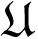
^⊥^ 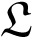 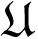
^⊥^*^T^* < 0,
- ∃*μ* ∈ **R***^n^*: 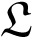 − *μ* 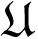 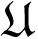
*^T^* < 0,
- ∃ 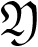 ∈ **R***^m^*^×^*^n^*: 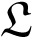 + 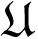 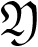 + 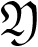
*^T^* 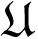
*^T^* < 0.

### Lemma 7

Given the matrices *Ẽ* and *F̃* with appropriate dimensions, then

$$\tilde{E}\Delta \tilde{F}+{\tilde{F}}^{T}\Delta^{T}{\tilde{E}}^{T}<0$$

where ΔΔ*^T^* ≤ *I*, if and only if there exist a scalar *ε* > 0 such that

$$\left[\begin{array}{cc} {ɛ}^{-\frac{1}{2}}{\tilde{F}}^{T} & {ɛ}^{\frac{1}{2}}\tilde{E} \end{array}\right]\;\left[\begin{array}{c} {ɛ}^{-\frac{1}{2}}\tilde{F}\\ {ɛ}^{\frac{1}{2}}{\tilde{E}}^{T} \end{array}\right]<0$$

## 3. Main Results

In this section, the reliable filtering problem proposed in the above section will be investigated.

### Lemma 8

Consider system (10) for *i* = 0, 1,…,*q* and a given scalar *γ*_1_ < 0, then Equation (16) holds, *i.e.*, system (10) is with a finite frequency *l*_2_ gain *γ*_1_,if there exist matrices ${\text{P}}_{\sigma_{k}i}={\text{P}}_{\sigma_{k}i}^{T},{ℛ}_{i}={ℛ}_{i}^{T}$ and ${\text{Q}}_{\sigma_{k}i}={\text{Q}}_{\sigma_{k}i}^{T}>0,\sigma_{k}\in \{1,2\}$ such that the following inequalities hold

(18) $${\left[\begin{array}{lll} {\text{A}}_{\sigma_{k}i} & {\text{A}}_{\sigma_{k}di} & {ℬ}_{w}\\ I & 0 & 0 \end{array}\right]}^{T}\;\Xi \left[\begin{array}{lll} {\text{A}}_{\sigma_{k}i} & {\text{A}}_{\sigma_{k}di} & {ℬ}_{w}\\ I & 0 & 0 \end{array}\right]+{\left[\begin{array}{lll} \text{C} & 0 & 0\\ 0 & 0 & I \end{array}\right]}^{T}\;\prod \;\left[\begin{array}{lll} \text{C} & 0 & 0\\ 0 & 0 & I \end{array}\right]\;+{\left[\begin{array}{lll} I & 0 & 0\\ 0 & I & 0 \end{array}\right]}^{T}\;\left[\begin{array}{cc} {ℛ}_{i} & 0\\ 0 & -{ℛ}_{i} \end{array}\right]\left[\begin{array}{lll} I & 0 & 0\\ 0 & I & 0 \end{array}\right]<0$$

where $\prod =\left[\begin{array}{cc} I & 0\\ 0 & -\gamma_{1}^{2}I \end{array}\right]$ *and*

- For the low-frequency range |*θ_w_*| ≤ *ϑ_wl_*
  (19) $$\Xi_{i}=\left[\begin{array}{cc} -{\text{P}}_{\sigma_{k+1}i} & {\text{Q}}_{\sigma_{k}i}\\ {\text{Q}}_{\sigma_{k}i} & {\text{P}}_{\sigma_{k}i}-2\cos\vartheta_{wl}{\text{Q}}_{\sigma_{k}i} \end{array}\right]$$
- For the middle-frequency range *ϑ*_1_ ≤ *θ_w_* ≤ *ϑ*_2_
  (20) $$\Xi_{i}=\left[\begin{array}{cc} -{\text{P}}_{\sigma_{k+1}i} & e^{j\vartheta_{c}}{\text{Q}}_{\sigma_{k}i}\\ e^{-j\vartheta_{c}}{\text{Q}}_{\sigma_{k}i} & {\text{P}}_{\sigma_{k}i}-2\cos\vartheta_{w}{\text{Q}}_{\sigma_{k}i} \end{array}\right]$$
  where *ϑ_c_* = (*ϑ*_2_ + *ϑ*_1_)/2, *ϑ_w_* = (*ϑ*_2_ − *ϑ*_1_)/2.
- For the high-frequency range |*θ_w_*| ≥ *ϑ_wh_*
  (21) $$\Xi_{i}=\left[\begin{array}{cc} -{\text{P}}_{\sigma_{k+1}i} & -{\text{Q}}_{\sigma_{k}i}\\ -{\text{Q}}_{\sigma_{k}i} & {\text{P}}_{\sigma_{k}i}-2\cos\vartheta_{wh}{\text{Q}}_{\sigma_{k}i} \end{array}\right]$$

#### Proof

We first consider the middle-frequency case for system (10) with *y_si_*(*k*) = 0. Assume Equation (14) holds, pre- and post-multiplying it by [*ξ^T^*(*k*) *s^T^*(*k*)] and its transpose, we can derive

(22) $$\begin{array}{l} \zeta^{T}(k)=({\text{P}}_{\sigma_{k}i}+R_{i})\zeta (k)-\zeta^{T}(k+1){\text{P}}_{\sigma_{k+1}i}\zeta (k+1)-\zeta^{T}(k-1)R_{i}\zeta (k-1)\\ +e^{T}(k)e(k)-\gamma_{1}^{2}w^{T}(k)w(k)\\ +tr\{{\text{Q}}_{\sigma_{k}i}(e^{j\vartheta_{c}}\zeta (k)\zeta^{T}(k+1)-e^{-j\vartheta_{c}}\zeta (k+1)\zeta^{T}(k)-2\cos\vartheta_{w}\zeta (k))\}\leq 0 \end{array}$$

Summing up Equation (22) from 0 to ∞ with respect to *k*, it is easy to obtain

(23) $$\sum_{k=0}^{\infty }\;\left(𝔼\left(e^{T}(k)e(k)-\gamma_{1}^{2}w^{T}(k)w(k)\right)\right)+tr\{{\text{Q}}_{\sigma_{k}i}\text{Z}\}\leq 0$$

since system (10) is asymptotical stable and *ξ*(0) = 0, where

(24) $$\text{Z}:=\sum_{k=0}^{\infty }\left(e^{j\vartheta_{c}}\zeta (k)\zeta^{T}(k+1)+e^{-j\vartheta_{c}}\zeta (k+1)\zeta^{T}(k)-2\cos\vartheta_{w}\zeta (k)\zeta^{T}(k)\right)$$

It is easy to prove that −*Z* is equal to the left-hand side of Equation (14), thus we have *Z* ≥ 0. Further, from *Q_σki_* > 0, one can obtain that the term *tr*{*Q_σki_Z*} ≥ 0 while Equation (14) is satisfied. Hence we have $\sum_{k=0}^{\infty }(𝔼(e^{T}(k)e(k)-\gamma_{1}^{2}w^{T}(k)w))\leq 0$, which is equivalent to the condition Equation (16) for middle-frequency in Definition 5.

Similarly, by choosing *ϑ*_1_:= −*ϑ_wl_*, *ϑ*_2_:= *ϑ_wl_* for low-frequency case and *ϑ*_1_:= *ϑ_wh_*, *ϑ*_2_:= 2*π* − *ϑ_wh_* for high-frequency case, respectively, the results for these two cases can be derived by following the same procedure of the above proof. This completes the proof.

### Remark 9

Lemma 8 presents an analysis condition for finite frequency *l*_2_ gain of system (10), where less conservatism is introduced compared with the existing full frequency conditions when frequency ranges of disturbances are known.

### Remark 10

The sufficient condition, which guarantees a prescribed low frequency *l*_2_ gain from y_si_(*k*) to *e*(*k*) for system (10), can be obtained by following the same process of Lemma 8 and utilizing relevant system matrices.

### 3.1. Finite Frequency Performance from w(k) to e(k)

In this section, sufficient conditions to capture the finite frequency performance from *w*(*k*) to *e*(*k*) for system (10) will be derived with the aid of Lemma 8.

#### Theorem 11

Consider system (10) in fault free and faulty cases (*i.e., i* = 0, 1,…,*q*) for given low frequency range |*θ*| ≤ *ϑ_wl_*, which is with a prescribed *l*_2_ gain *γ*_1_ from *w*(*k*) to *e*(*k*),*i.e.*, the condition (16) holds if there exist a scalar *ε*_1_ > 0, matrices *X, Y, N, A_f_, ℬ_f_, C_f_* and

$$\begin{array}{ll} {\text{P}}_{\iota i}^{T}={\text{P}}_{\iota i}=\left[\begin{array}{cc} {\text{P}}_{\iota i1} & *\\ {\text{P}}_{\iota i2} & {\text{P}}_{\iota i3} \end{array}\right]\;, & {\text{P}}_{\kappa i}^{T}={\text{P}}_{\kappa i}=[\begin{array}{cc} {\text{P}}_{\kappa i1} & *\\ {\text{P}}_{\kappa i2} & {\text{P}}_{\kappa i3} \end{array}]\;,\\ {ℛ}_{i}^{T}={ℛ}_{i}=\left[\begin{array}{cc} {ℛ}_{i1} & *\\ {ℛ}_{i2} & {ℛ}_{i3} \end{array}\right]\;, & {\text{Q}}_{\kappa i}^{T}={\text{Q}}_{\kappa i}=\left[\begin{array}{cc} {\text{Q}}_{\kappa i1} & *\\ {\text{Q}}_{\kappa i2} & {\text{Q}}_{\kappa i3} \end{array}\right]>0 \end{array}$$

with *ι, κ ∈* {1, 2} such that the following conditions hold

(25) Ψ<0

where

$$\Psi =\left[\begin{array}{ccccccccc} -{\text{P}}_{\iota i1} & * & * & * & * & * & * & * & *\\ -{\text{P}}_{\iota i2} & -{\text{P}}_{\iota i3} & * & * & * & * & * & * & *\\ {\text{Q}}_{\kappa i1}-\text{X} & {\text{Q}}_{\kappa i2}^{T}-\text{N} & \Psi_{3}^{3} & * & * & * & * & * & *\\ {\text{Q}}_{\kappa i2}-\text{Y} & {\text{Q}}_{\kappa i3}-\text{N} & \Psi_{4}^{3} & \Psi_{4}^{4} & * & * & * & * & *\\ 0 & 0 & \Psi_{5}^{3} & \Psi_{5}^{4} & \Psi_{5}^{5} & * & * & * & *\\ 0 & 0 & 0 & 0 & -{ℛ}_{i2} & -{ℛ}_{i3} & * & * & *\\ 0 & 0 & B^{T}{\text{X}}^{T} & B^{T}{\text{Y}}^{T} & 0 & 0 & -\gamma_{1}^{2}I & * & *\\ 0 & 0 & E & -C_{f} & 0 & 0 & 0 & -I & *\\ 0 & 0 & {ℬ}_{f}^{T} & {ℬ}_{f}^{T} & 0 & 0 & 0 & 0 & -{ɛ}_{1}I \end{array}\right]$$

and

$$\begin{array}{l} \Psi_{3}^{3}(\kappa =1)={\text{P}}_{\kappa i1}-2\cos\vartheta_{wl}{\text{Q}}_{\kappa i1}+{ℛ}_{i1}+\delta_{q}^{2}{ɛ}_{1}C^{T}F_{i}^{T}F_{i}C+He(\text{X}A+{ℬ}_{f}F_{i}C),\\ \Psi_{3}^{3}(\kappa =2)={\text{P}}_{\kappa i1}-2\cos\vartheta_{wl}{\text{Q}}_{\kappa i1}+{ℛ}_{i1}+He(\text{X}A),\\ \Psi_{4}^{3}(\kappa =1)={\text{P}}_{\kappa i2}-2\cos\vartheta_{wl}{\text{Q}}_{\kappa i2}+{ℛ}_{i2}+{ℬ}_{f}F_{i}C+{\text{A}}_{f}^{T}+\text{Y}A,\\ \Psi_{4}^{3}(\kappa =2)={\text{P}}_{\kappa i2}-2\cos\vartheta_{wl}{\text{Q}}_{\kappa i2}+{ℛ}_{i2}+{\text{A}}_{f}^{T}+\text{Y}A,\\ \Psi_{4}^{4}={\text{P}}_{\kappa i3}-2\cos\vartheta_{wl}{\text{Q}}_{\kappa i3}+{ℛ}_{i3}+He({\text{A}}_{f}),\\ \begin{array}{ll} \Psi_{5}^{3}(\kappa =1)=\Omega_{5}^{4}(\kappa =1)=0, & \Psi_{5}^{3}(\kappa =1)=\Omega_{5}^{4}(\kappa =1)=C^{T}F_{i}^{T}{ℬ}_{f}^{T}\\ \Psi_{5}^{5}(\kappa =1)=-{ℛ}_{i1}, & \Psi_{5}^{5}(\kappa =1)=-{ℛ}_{i1}+\delta_{q}^{1}{ɛ}_{1}C^{T}F_{i}^{T}F_{i}C \end{array} \end{array}$$

##### Proof

It is shown, from Lemma 8, that the condition (16) can be reached if Equation (18) holds. Further, the inequality (18) can be rewritten to

(26) $$J\Delta J^{T}<0$$

where $J=\left[\begin{array}{llll} {\text{A}}_{\kappa i}^{T} & I & 0 & 0\\ {\text{A}}_{\kappa di}^{T} & 0 & I & 0\\ {ℬ}_{w}^{T} & 0 & 0 & I \end{array}\right]$ and

$$\Delta =\left[\begin{array}{cccc} -{\text{P}}_{\iota i} & * & * & *\\ {\text{Q}}_{\kappa i} & {\text{P}}_{\kappa i}-2\cos\vartheta_{wl}{\text{Q}}_{\kappa i}+{ℛ}_{i}+{\text{C}}^{T}\text{C} & * & *\\ 0 & 0 & -{ℛ}_{i} & *\\ 0 & 0 & 0 & -\gamma_{1}^{2}I \end{array}\right]$$

Exploiting Lemma 6 and explicit null space bases calculations on Equation (26), it is easy to get that Equation (26) holds if and only if

(27) $$\Delta +He\left(J^{⊥}[\begin{array}{cccc} 0 & {\text{W}}^{T} & 0 & 0 \end{array}]\right)<0$$

that is

$$\left[\begin{array}{cccc} -{\text{P}}_{\iota i} & * & * & *\\ {\text{Q}}_{\kappa i}-\text{W} & {\text{A}}_{\kappa i}-2\cos\vartheta_{wl}{\text{Q}}_{\kappa i}+{ℛ}_{i}+{\text{C}}^{T}\text{C}+He(\text{W}{\text{A}}_{\kappa i}) & * & *\\ 0 & {\text{A}}_{d\kappa i}^{T}{\text{W}}^{T} & -{ℛ}_{i} & *\\ 0 & {ℬ}_{w}^{T}{\text{W}}^{T} & 0 & -\gamma_{1}^{2}I \end{array}\right]<0$$

where *W* is a matrix variable introduced by Lemma 6 and *J*^⊥^ = [−*I A_κi_* ℬ*_w_A_κdi_*]*^T^* is utilized.

Performing Schur's complement on Equation (27) yields to that

$$\left[\begin{array}{ccccc} -{\text{P}}_{\iota i} & * & * & * & *\\ {\text{Q}}_{\kappa i}-\text{W} & {\text{P}}_{\kappa i}-2\cos\vartheta_{wl}{\text{Q}}_{\kappa i}+{ℛ}_{i}+He(\text{W}{\text{A}}_{\kappa i}) & * & * & *\\ 0 & {\text{A}}_{d\kappa i}^{T}{\text{W}}^{T} & -{ℛ}_{i} & * & *\\ 0 & {ℬ}_{w}^{T}{\text{W}}^{T} & 0 & -\gamma_{1}^{2}I & *\\ 0 & \text{C} & 0 & 0 & -I \end{array}\right]<0$$

Partitioning *W* as the following form

(28) $$\text{W}=\left[\begin{array}{cc} \text{X} & \text{N}\\ \text{Y} & \text{N} \end{array}\right]$$

defining the following new variables

(29) $$\begin{array}{cc} {\text{A}}_{f}=\text{N}A_{f}, & {ℬ}_{f}= \end{array}\text{N}B_{f}$$

and applying Lemma 7 on Equations (27), (25) can be reached easily. This proof is completed.

#### Remark 12

In Theorem 11, by introducing a variable *W*, the coupling between the Lyapunov matrices and the filter gains will be eliminated, which does not present any structural constraint.

The previous Theorem 11 presented the condition to capture the low frequency performance. Similarly, conditions for middle frequency and high frequency performance are presented in the following two corollaries.

#### Corollary 13

Consider system (10) in fault free and faulty cases (*i.e., i* = 0, 1,…,*q*) for given middle frequency range *ϑ*_1_ ≤ |*θ_w_*| ≤ *ϑ*_2_,which is with a prescribed *l*_2_ gain *γ*_1_ from *w*(*k*) to *e*(*k*),*i.e.*, the condition (16) holds if there exist a scalar *ε*_1_ > 0, matrices *X, Y, N, A_f_, ℬ_f_, C_f_* and

$$\begin{array}{cc} {\text{P}}_{\iota i}^{T}={\text{P}}_{\iota i}=\left[\begin{array}{cc} {\text{P}}_{\iota i1} & *\\ {\text{P}}_{\iota i2} & {\text{P}}_{\iota i3} \end{array}\right]\;, & {\text{P}}_{\kappa i}^{T}={\text{P}}_{\kappa i}=\left[\begin{array}{cc} {\text{P}}_{\kappa i1} & *\\ {\text{P}}_{\kappa i2} & {\text{P}}_{\kappa i3} \end{array}\right]\;,\\ {ℛ}_{i}^{T}={ℛ}_{i}=\left[\begin{array}{cc} {ℛ}_{i1} & *\\ {ℛ}_{i2} & {ℛ}_{i3} \end{array}\right]\;, & {\text{Q}}_{\kappa i}^{T}={\text{Q}}_{\kappa i}=\left[\begin{array}{cc} {\text{Q}}_{\kappa i1} & *\\ {\text{Q}}_{\kappa i2} & {\text{Q}}_{\kappa i3} \end{array}\right]>0 \end{array}$$

with *ι, κ ∈* {1, 2} such that the following conditions hold

(30) Ψ<0

where

$$\Psi =\left[\begin{array}{ccccccccc} -{\text{P}}_{\iota i1} & * & * & * & * & * & * & * & *\\ -{\text{P}}_{\iota i2} & -{\text{P}}_{\iota i3} & * & * & * & * & * & * & *\\ e^{-j\vartheta_{c}}{\text{Q}}_{\kappa i1}-\text{X} & e^{-j\vartheta_{c}}{\text{Q}}_{\kappa i2}^{T}-\text{N} & \Psi_{3}^{3} & * & * & * & * & * & *\\ e^{-j\vartheta_{c}}{\text{Q}}_{\kappa i2}-\text{Y} & e^{-j\vartheta_{c}}{\text{Q}}_{\kappa i3}-\text{N} & \Psi_{4}^{3} & \Psi_{4}^{4} & * & * & * & * & *\\ 0 & 0 & \Psi_{5}^{3} & \Psi_{5}^{4} & \Psi_{5}^{5} & * & * & * & *\\ 0 & 0 & 0 & 0 & -{ℛ}_{i2} & -{ℛ}_{i3} & * & * & *\\ 0 & 0 & B^{T}{\text{X}}^{T} & B^{T}{\text{Y}}^{T} & 0 & 0 & -\gamma_{1}^{2}I & * & *\\ 0 & 0 & E & -C_{f} & 0 & 0 & 0 & -I & *\\ 0 & 0 & {ℬ}_{f}^{T} & {ℬ}_{f}^{T} & 0 & 0 & 0 & 0 & -{ɛ}_{1}I \end{array}\right]$$

and *ϑ_c_* = (*ϑ*_2_ + *ϑ*_1_)/2, *ϑ_w_* = (*ϑ*_2_ − *ϑ*_1_)/2,

$$\begin{array}{l} \Psi_{3}^{3}(\kappa =1)={\text{P}}_{\kappa i1}-2\cos\vartheta_{w}{\text{Q}}_{\kappa i1}+{ℛ}_{i1}+\delta_{q}^{2}{ɛ}_{1}C^{T}F_{i}^{T}F_{i}C+He(\text{X}A+{ℬ}_{f}F_{i}C),\\ \Psi_{3}^{3}(\kappa =2)={\text{P}}_{\kappa i1}-2\cos\vartheta_{w}{\text{Q}}_{\kappa i1}+{ℛ}_{i1}+He(\text{X}A),\\ \Psi_{4}^{3}(\kappa =1)={\text{P}}_{\kappa i2}-2\cos\vartheta_{w}{\text{Q}}_{\kappa i2}+{ℛ}_{i2}+{ℬ}_{f}F_{i}C+{\text{A}}_{f}^{T}+\text{Y}A,\\ \Psi_{4}^{3}(\kappa =2)={\text{P}}_{\kappa i2}-2\cos\vartheta_{w}{\text{Q}}_{\kappa i2}+{ℛ}_{i2}+{\text{A}}_{f}^{T}+\text{Y}A,\\ \Psi_{4}^{4}={\text{P}}_{\kappa i3}-2\cos\vartheta_{l}{\text{Q}}_{\kappa i3}+{ℛ}_{i3}+He({\text{A}}_{f}),\\ \begin{array}{ll} \Psi_{5}^{3}(\kappa =1)=\Omega_{5}^{4}(\kappa =1)=0, & \Psi_{5}^{3}(\kappa =2)=\Omega_{5}^{4}(\kappa =2)=C^{T}F_{i}^{T}{ℬ}_{f}^{T}\\ \Psi_{5}^{5}(\kappa =1)=-{ℛ}_{i1}, & \Psi_{5}^{5}(\kappa =2)=-{ℛ}_{i1}+\delta_{q}^{1}{ɛ}_{1}C^{T}F_{i}^{T}F_{i}C \end{array} \end{array}$$

##### Proof

By following the same lines of Theorem 1, it is immediate.

#### Corollary 14

Consider system (10) in fault-free and faulty cases (*i.e., i* = 0, 1,…, *q*) for given high frequency range |*θ_w_*| ≥ *ϑ_wh_*, which is with a prescribed *l*_2_ gain *γ*_1_ from *w*(*k*) to *e*(*k*),*i.e.*, the condition (16) holds if there exist a scalar *ε*_1_ > 0,matrices *X, Y, N, A_f_, ℬ_f_, C_f_* and

$$\begin{array}{cc} {\text{P}}_{\iota i}^{T}={\text{P}}_{\iota i}=\left[\begin{array}{cc} {\text{P}}_{\iota i1} & *\\ {\text{P}}_{\iota i2} & {\text{P}}_{\iota i3} \end{array}\right]\;, & {\text{P}}_{\kappa i}^{T}={\text{P}}_{\kappa i}=\left[\begin{array}{cc} {\text{P}}_{\kappa i1} & *\\ {\text{P}}_{\kappa i2} & {\text{P}}_{\kappa i3} \end{array}\right]\;,\\ {ℛ}_{i}^{T}={ℛ}_{i}=\left[\begin{array}{cc} {ℛ}_{i1} & *\\ {ℛ}_{i2} & {ℛ}_{i3} \end{array}\right]\;, & {\text{Q}}_{\kappa i}^{T}={\text{Q}}_{\kappa i}=\left[\begin{array}{cc} {\text{Q}}_{\kappa i1} & *\\ {\text{Q}}_{\kappa i2} & {\text{Q}}_{\kappa i3} \end{array}\right]>0 \end{array}$$

with *ι, κ* ∈ {1, 2} such that the following conditions hold

(31) Ψ<0

where

$$\Psi =\left[\begin{array}{ccccccccc} -{\text{P}}_{\iota i1} & * & * & * & * & * & * & * & *\\ -{\text{P}}_{\iota i2} & -{\text{P}}_{\iota i3} & * & * & * & * & * & * & *\\ -{\text{Q}}_{\kappa i1}-\text{X} & -{\text{Q}}_{\kappa i2}^{T}-\text{N} & \Psi_{3}^{3} & * & * & * & * & * & *\\ -{\text{Q}}_{\kappa i2}-\text{Y} & -{\text{Q}}_{\kappa i3}-\text{N} & \Psi_{4}^{3} & \Psi_{4}^{4} & * & * & * & * & *\\ 0 & 0 & \Psi_{5}^{3} & \Psi_{5}^{4} & \Psi_{5}^{5} & * & * & * & *\\ 0 & 0 & 0 & 0 & -{ℛ}_{i2} & -{ℛ}_{i3} & * & * & *\\ 0 & 0 & B^{T}{\text{X}}^{T} & B^{T}{\text{Y}}^{T} & 0 & 0 & -\gamma_{1}^{2}I & * & *\\ 0 & 0 & E & -C_{f} & 0 & 0 & 0 & -I & *\\ 0 & 0 & {ℬ}_{f}^{T} & {ℬ}_{f}^{T} & 0 & 0 & 0 & 0 & -{ɛ}_{1}I \end{array}\right]$$

and

$$\begin{array}{l} \Psi_{3}^{3}(\kappa =1)={\text{P}}_{\kappa i1}-2\cos\vartheta_{wh}{\text{Q}}_{\kappa i1}+{ℛ}_{i1}+\delta_{q}^{2}{ɛ}_{1}C^{T}F_{i}^{T}F_{i}C+He(\text{X}A+{ℬ}_{f}F_{i}C),\\ \Psi_{3}^{3}(\kappa =2)={\text{P}}_{\kappa i1}-2\cos\vartheta_{wh}{\text{Q}}_{\kappa i1}+{ℛ}_{i1}+He(\text{X}A),\\ \Psi_{4}^{3}(\kappa =1)={\text{P}}_{\kappa i2}-2\cos\vartheta_{wh}{\text{Q}}_{\kappa i2}+{ℛ}_{i2}+{ℬ}_{f}F_{i}C+{\text{A}}_{f}^{T}+\text{Y}A,\\ \Psi_{4}^{3}(\kappa =2)={\text{P}}_{\kappa i2}-2\cos\vartheta_{wh}{\text{Q}}_{\kappa i2}+{ℛ}_{i2}+{\text{A}}_{f}^{T}+\text{Y}A,\\ \Psi_{4}^{4}={\text{P}}_{\kappa i3}-2\cos\vartheta_{l}{\text{Q}}_{\kappa i3}+{ℛ}_{i3}+He({\text{A}}_{f}),\\ \begin{array}{ll} \Psi_{5}^{3}(\kappa =1)=\Omega_{5}^{4}(\kappa =1)=0, & \Psi_{5}^{3}(\kappa =2)=\Omega_{5}^{4}(\kappa =2)=C^{T}F_{i}^{T}{ℬ}_{f}^{T}\\ \Psi_{5}^{5}(\kappa =1)=-{ℛ}_{i1}, & \Psi_{5}^{5}(\kappa =2)=-{ℛ}_{i1}+\delta_{q}^{1}{ɛ}_{1}C^{T}F_{i}^{T}F_{i}C \end{array} \end{array}$$

##### Proof

By following the same lines of Theorem 1, it is immediate.

### 3.2. Low Frequency Performance from y_si_(k) to e(k)

In this subsection, sufficient conditions to capture the low frequency performance (17) for system (10) will be deduced.

#### Theorem 15

Consider system (10) in faulty cases (*i.e., i* = 1,…, *q*) for given low frequency range |*θ_s_*| ≤ *ϑ_sl_*, which is with a prescribed low frequency *l*_2_ gain *γ*_2_ for nonzero *y_si_*(*k*),*i.e.*, condition (17) holds, if there exist a scalar *ε*_2_ > 0, matrices *X, Y, N, A_f_, ℬ_f_, C_f_* and

$$\begin{array}{cc} {\text{P}}_{s\iota i}^{T}={\text{P}}_{s\iota i}=\left[\begin{array}{cc} {\text{P}}_{s\iota i1} & *\\ {\text{P}}_{s\iota i2} & {\text{P}}_{s\iota i3} \end{array}\right]\;, & {\text{P}}_{s\kappa i}^{T}={\text{P}}_{s\kappa i}=\left[\begin{array}{cc} {\text{P}}_{s\kappa i1} & *\\ {\text{P}}_{s\kappa i2} & {\text{P}}_{s\kappa i3} \end{array}\right]\;,\\ {ℛ}_{si}^{T}={ℛ}_{si}=\left[\begin{array}{cc} {ℛ}_{si1} & *\\ {ℛ}_{si2} & {ℛ}_{si3} \end{array}\right]\;, & {\text{Q}}_{s\kappa i}^{T}={\text{Q}}_{s\kappa i}=\left[\begin{array}{cc} {\text{Q}}_{s\kappa i1} & *\\ {\text{Q}}_{s\kappa i2} & {\text{Q}}_{s\kappa i3} \end{array}\right]>0 \end{array}$$

with *ι, κ ∈* {1, 2} such that the following conditions hold

(32) Ω<0

where

$$\Omega =\left[\begin{array}{ccccccccc} -{\text{P}}_{s\iota i1} & * & * & * & * & * & * & * & *\\ -{\text{P}}_{s\iota i2} & -{\text{P}}_{\iota i3} & * & * & * & * & * & * & *\\ {\text{Q}}_{s\kappa i1}-\text{X} & {\text{Q}}_{s\kappa i2}^{T}-\text{N} & \Omega_{3}^{3} & * & * & * & * & * & *\\ {\text{Q}}_{s\kappa i2}-\text{Y} & {\text{Q}}_{s\kappa i3}-\text{N} & \Omega_{4}^{3} & \Omega_{4}^{4} & * & * & * & * & *\\ 0 & 0 & \Omega_{5}^{3} & \Omega_{5}^{4} & \Omega_{5}^{5} & * & * & * & *\\ 0 & 0 & 0 & 0 & -{ℛ}_{si2} & -{ℛ}_{si3} & * & * & *\\ 0 & 0 & {\left(I-F_{i}\right)}^{T}{ℬ}_{f}^{T} & {\left(I-F_{i}\right)}^{T}{ℬ}_{f}^{T} & 0 & 0 & \Omega_{7}^{7} & * & *\\ 0 & 0 & E & -C_{f} & 0 & 0 & 0 & -I & *\\ 0 & 0 & {ℬ}_{f}^{T} & {ℬ}_{f}^{T} & 0 & 0 & 0 & 0 & -{ɛ}_{2}I \end{array}\right]$$

and

$$\begin{array}{l} \Omega_{3}^{3}(\kappa =1)={\text{P}}_{s\kappa i1}-2\cos\vartheta_{sl}{\text{Q}}_{s\kappa i1}+{ℛ}_{si1}+\delta_{q}^{2}{ɛ}_{2}C^{T}F_{i}^{T}F_{i}C+He(\text{X}A+{ℬ}_{f}F_{i}C),\\ \Omega_{3}^{3}(\kappa =1)={\text{P}}_{s\kappa i1}-2\cos\vartheta_{sl}{\text{Q}}_{s\kappa i1}+{ℛ}_{si1}+He(\text{X}A),\\ \Omega_{4}^{3}(\kappa =1)={\text{P}}_{s\kappa i2}-2\cos\vartheta_{sl}{\text{Q}}_{s\kappa i2}+{ℛ}_{si2}+{ℬ}_{f}F_{i}C+{\text{A}}_{f}^{T}+\text{Y}A,\\ \Omega_{4}^{3}(\kappa =2)={\text{P}}_{s\kappa i2}-2\cos\vartheta_{sl}{\text{Q}}_{s\kappa i2}+{ℛ}_{si2}+{\text{A}}_{f}^{T}+\text{Y}A,\\ \Omega_{4}^{4}={\text{P}}_{s\kappa i3}-2\cos\vartheta_{l}{\text{Q}}_{s\kappa i3}+{ℛ}_{si3}+He({\text{A}}_{f}),\\ \begin{array}{ll} \Omega_{5}^{3}(\kappa =1)=0, & \Omega_{5}^{3}(\kappa =2)=C^{T}F_{i}^{T}{ℬ}_{f}^{T}\\ \Omega_{5}^{5}(\kappa =1)=-{ℛ}_{si1}, & \Omega_{5}^{5}(\kappa =2)=-{ℛ}_{si1}+\delta_{q}^{2}{ɛ}_{2}C^{T}F_{i}^{T}F_{i}C \end{array}\\ \Omega_{7}^{7}=-\gamma_{2}^{2}I+\delta_{q}^{2}{ɛ}_{2}{\left(I-F_{i}\right)}^{T}(I-F_{i}), \end{array}$$

##### Proof

It is easily derived, from Lemma 8 and Remark 10, that the condition (17) holds if

(33) $${\left[\begin{array}{lll} {\text{A}}_{\sigma_{k}i} & {\text{A}}_{\sigma_{k}di} & {ℬ}_{si}\\ I & 0 & 0 \end{array}\right]}^{T}\Xi_{d}\;\left[\begin{array}{lll} {\text{A}}_{\sigma_{k}i} & {\text{A}}_{\sigma_{k}di} & {ℬ}_{si}\\ I & 0 & 0 \end{array}\right]+{\left[\begin{array}{lll} \text{C} & 0 & 0\\ 0 & 0 & I \end{array}\right]}^{T}\prod \;\left[\begin{array}{lll} \text{C} & 0 & 0\\ 0 & 0 & I \end{array}\right]+{\left[\begin{array}{lll} I & 0 & 0\\ 0 & I & 0 \end{array}\right]}^{T}\left[\begin{array}{cc} {ℛ}_{si} & 0\\ 0 & -{ℛ}_{si} \end{array}\right]\left[\begin{array}{lll} I & 0 & 0\\ 0 & I & 0 \end{array}\right]<0$$

where Ξ*_d_* has the similar structure as in Lemma 8 for low frequency case.

Following the same process in Theorem 11, we first rewrite the inequality (33) to the following form,

(34) $$𝕷\Theta {𝕷}^{T}<0$$

where $𝕷=\left[\begin{array}{llll} {\text{A}}_{\kappa i}^{T} & I & 0 & 0\\ {\text{A}}_{\kappa di}^{T} & 0 & I & 0\\ {ℬ}_{fi}^{T} & 0 & 0 & I \end{array}\right]$ and

$$\Theta =\left[\begin{array}{cccc} -{\text{P}}_{s\iota i} & {\text{Q}}_{s\kappa i} & * & *\\ {\text{Q}}_{s\kappa i} & {\text{P}}_{s\kappa i}-2\cos\vartheta_{sl}{\text{Q}}_{s\kappa i}+{ℛ}_{si}+{\text{C}}^{T}\text{C} & * & *\\ 0 & 0 & -{ℛ}_{si} & *\\ 0 & 0 & 0 & -\gamma_{2}^{2}I \end{array}\right]$$

Exploiting Lemma 6 and explicit null space bases calculations on it, we have Equation (34) is equivalent to

(35) $$\Xi +He\left({𝕷}^{⊥}[\begin{array}{cccc} 0 & {\text{W}}^{T} & 0 & 0 \end{array}]\right)<0$$

that is

$$\left[\begin{array}{ccccc} -{\text{P}}_{s\iota i} & * & * & * & *\\ {\text{Q}}_{\kappa i}-\text{W} & {\text{P}}_{s\kappa i}-2\cos\vartheta_{sl}{\text{Q}}_{s\kappa i}+{ℛ}_{si}+He(\text{W}{\text{A}}_{\kappa i}) & * & * & *\\ 0 & {\text{A}}_{\kappa di}^{T}{\text{W}}^{T} & -{ℛ}_{i} & * & *\\ 0 & {ℬ}_{fi}^{T}{\text{W}}^{T} & 0 & -\gamma_{2}^{2}I & *\\ 0 & \text{C} & 0 & 0 & -I \end{array}\right]<0$$

where *W* is a matrix variable introduced by Lemma 8 and 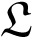
^⊥^ = [−*I A_κi_A_κdi_* ℬ*_fi_*] is utilized.

By applying Lemma 7 on Equation (35), the inequality Equation (32) can be reached easily This proof is completed.

### 3.3. Stability Condition

Since Theorems 1 and 2 presented in the above section cannot guarantee the stability of the system (10), in this subsection, asymptotical stability conditions for system (10) will be presented.

#### Theorem 16

Consider system (10) in fault free and faulty cases (*i.e., i* = 0, 1,…, *q*), it is asymptotical stable when *w*(*k*) = 0 and *y_si_*(*k*) = 0,if there exist a scalar *ε*_3_ > 0,matrices *X, Y, N, A_f_, ℬ_f_, C_f_* and

$$\begin{array}{c} {\text{P}}_{a\iota i}^{T}={\text{P}}_{a\iota i}=\left[\begin{array}{cc} {\text{P}}_{a\iota i1} & *\\ {\text{P}}_{a\iota i2} & {\text{P}}_{a\iota i3} \end{array}\right]>0\\ {\text{P}}_{a\kappa i}^{T}={\text{P}}_{a\kappa i}=\left[\begin{array}{cc} {\text{P}}_{a\kappa i1} & *\\ {\text{P}}_{a\kappa i2} & {\text{P}}_{a\kappa i3} \end{array}\right]>0\\ R_{ai}^{T}=R_{a}i=\left[\begin{array}{cc} R_{ai1} & *\\ R_{ai2} & R_{ai3} \end{array}\right]>0 \end{array}$$

with *ι, κ ∈* {1, 2} such that the following conditions hold

(36) Φ<0

where

$$\Phi =\left[\begin{array}{ccccccc} {\text{P}}_{a\iota i1}-He(\text{X}) & * & * & * & * & * & *\\ {\text{P}}_{a\iota i2}-\text{Y}-{\text{N}}^{T} & {\text{P}}_{a\iota i3}-He(\text{X}) & * & * & * & * & *\\ \Phi_{3}^{1} & \Phi_{3}^{2} & \Phi_{3}^{3} & * & * & * & *\\ {\text{A}}_{f}^{T} & {\text{A}}_{f}^{T} & -{\text{P}}_{a\kappa i2}+{ℛ}_{ai2} & -{\text{P}}_{a\kappa i3}+{ℛ}_{ai3} & * & * & *\\ \Phi_{5}^{1} & \Phi_{5}^{2} & 0 & 0 & \Phi_{5}^{5} & * & *\\ 0 & 0 & 0 & 0 & -{ℛ}_{ai2} & -{ℛ}_{ai3} & *\\ {ℬ}_{f}^{T} & {ℬ}_{f}^{T} & 0 & 0 & 0 & 0 & -{ɛ}_{3}I \end{array}\right]$$

and

$$\begin{array}{ll} \Phi_{3}^{1}(\kappa =1)={\text{A}}^{T}{\text{X}}^{T}+C^{T}F_{i}^{T}{ℬ}_{f}^{T}, & \Phi_{3}^{1}(\kappa =2)=A^{T}{\text{X}}^{T},\\ \Phi_{3}^{2}(\kappa =1)=A^{T}{\text{Y}}^{T}+C^{T}F_{i}^{T}{ℬ}_{f}^{T}, & \Phi_{3}^{2}(\kappa =2)=A^{T}{\text{Y}}^{T},\\ \Phi_{3}^{3}(\kappa =1)=-{\text{P}}_{a\kappa i1}+{ℛ}_{ai1}+\delta_{q}^{2}{ɛ}_{3}C^{T}F_{i}^{T}F_{i}C, & \Phi_{3}^{3}(\kappa =2)-{\text{P}}_{a\kappa i1}+{ℛ}_{ai1},\\ \Phi_{5}^{1}(\kappa =1)=\Phi_{5}^{2}(\kappa =1)=0, & \Phi_{5}^{1}(\kappa =2)=\Phi_{5}^{2}(\kappa =2)=C^{T}F_{i}^{T}{ℬ}_{f}^{T},\\ \Phi_{5}^{5}(\kappa =1)=-{ℛ}_{ai1}, & \Phi_{5}^{5}(\kappa =2)=-{ℛ}_{ai1}+\delta_{q}^{2}{ɛ}_{3}C^{T}F_{i}^{T}F_{i}C. \end{array}$$

##### Proof

Consider the following Lyapunov functional candidate for *w*(*k*) = 0 and *y_si_*(*k*) = 0

(37) $$V_{\kappa i}(k)=\zeta^{T}(k){\text{P}}_{a\kappa i}\zeta (k)+\zeta^{T}(k-1){ℛ}_{ai}\zeta (k-1)$$

The forward difference of the Lyapunov functional *V_i_*(*k*) along the trajectories of the system (10) is given by

(38) $$\begin{array}{cc} \Delta V_{\kappa i}(k) & =V_{\iota i}(k+1)-V_{\kappa i}(k)\\ & =\zeta^{T}(k)\left({\text{A}}_{\kappa i}^{T}{\text{P}}_{a\iota i}{\text{A}}_{\kappa i}-{\text{P}}_{a\iota i}+{ℛ}_{ai}\right)\zeta (k)+\zeta^{T}(k)({\text{A}}_{\kappa i}^{T}{\text{P}}_{a\iota i}{\text{A}}_{\kappa di})\zeta (k-1)+\zeta^{T}(k-1)({\text{A}}_{\kappa di}^{T}{\text{P}}_{a\iota i}{\text{A}}_{\kappa i})\\ & =\xi^{T}(k){ϒ}_{\kappa i}\xi (k) \end{array}\zeta (k)+\zeta^{T}(k-1)({\text{A}}_{\kappa di}^{T}{\text{P}}_{a\iota i}{\text{A}}_{\kappa di}-{ℛ}_{ai})\zeta (k-1)$$

where *ζ*(*k*) = [*ζ^T^* (*k*) *ζ^T^*(*k* − 1)]*^T^* and

$${ϒ}_{\kappa i}=\left[\begin{array}{cc} {\text{A}}_{\kappa i}^{T}{\text{P}}_{a\iota i}{\text{A}}_{\kappa i}-{\text{P}}_{a\iota i}+{ℛ}_{ai} & *\\ {\text{A}}_{\kappa di}^{T}{\text{P}}_{a\iota i}{\text{A}}_{\kappa i} & {\text{A}}_{\kappa i}^{T}{\text{P}}_{a\iota i}{\text{A}}_{\kappa di}-{ℛ}_{ai} \end{array}\right]$$

By following an opposite direction to the proof for Theorem 11 and exploiting Schur's complement and Lemma 7 on Equation (36), we have *γ_κi_* < 0, which implies that Δ*V_κi_*(*k*) < 0. Therefore, from Equation (38), one can easily obtain, for a sufficiently small scalar *ρ* > 0 and *ζ*(*k*) ≠ 0, that

(39) $$\Delta V_{ki}(k)<-\rho {\left\|\varsigma (k)\right\|}^{2}$$

Then, from Definition 4, the asymptotical stability of system (10) can be established. This proof is completed.

### 3.4. Algorithm

Based on the above analysis, a set of optimal solutions *A_f_, ℬ_f_* and *C_f_* can be obtained by solving the following optimization problem for given *δ_q_*:

(40) $$\begin{array}{lll} & \min & a\gamma_{1}+b\gamma_{2}\\ s.t. & (25), & \;\text{for}\;i=0,1,\ldots ,q,\\ & (32), & \;\text{for}\;i=1,2,\ldots ,q,\\ & (36), & \;\text{for}\;i=0,1,\ldots ,q, \end{array}$$

where *a* and *b* are weighting factors.

Then the dynamic output feedback controller gains can be computed by the following equalities:

(41) $$\begin{array}{lll} A_{f}={\text{N}}^{-1}{\text{A}}_{f}, & B_{f}={\text{N}}^{-1}{ℬ}_{f}, & C_{f}=C_{f} \end{array}$$

#### Remark 17

On the other hand, we can obtain a coarser quantizer through solving the following optimization problem for given finite-frequency *l*_2_ gains *γ*_2_ and *γ*_2_

(42) $$\begin{array}{lll} & \max & \delta_{q}\\ s.t. & (25), & \text{for}\;i=0,1,\ldots ,q\\ & (32), & \text{for}\;i=1,2,\ldots ,q\\ & (36), & \text{for}\;i=0,1,\ldots ,q \end{array}$$

## 4. Example

In this section, an application and simulations are given to illustrate the effectiveness of the proposed methods.

The utilized model is the F-404 aircraft engine described by the following state space model [31],

(43) $$\begin{array}{c} \dot{x}(t)=\left[\begin{array}{rrr} -1.4600 & 0 & 2.4280\\ 0.1643 & -0.4000 & -0.3788\\ 0.3107 & 0 & -2.2300 \end{array}\right]\;x(t)+\left[\begin{array}{r} -0.09\\ -0.14\\ 0.02 \end{array}\right]\;w(t)\\ y(t)=\left[\begin{array}{rrr} 1 & 1 & 0\\ 0 & 1 & 1 \end{array}\right]\;x(t)\\ z(t)=\left[\begin{array}{rrr} 0.2 & -0.7 & 0.5\\ 0.3 & 0.6 & -0.4 \end{array}\right]\;x(t) \end{array}$$

**Figure 2.**
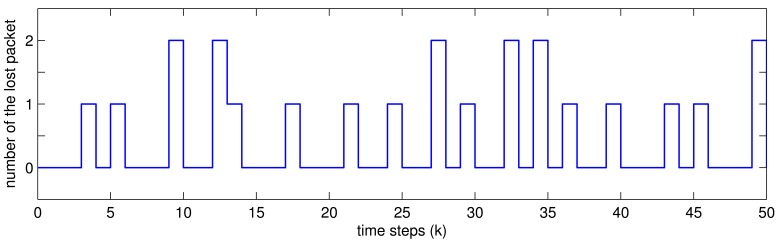
The number of the lost packet.

Assume the sampling period is *h* = 1*s*, and packet transmission is as shown in Figure 2, which is subject to the rate of packet lost 1 − *δ̄* = 0.32.

For given *ϑ_wl_* = 0.2, *ϑ_sl_* = 0 and *δ_q_* = 0.6, solving the optimization problem (40), we can obtain the optimal value for low frequency performances are, respectively, *γ*_1_ = 0.0825 and *γ*_2_ = 0.0378 with the corresponding reliable filter parameters

(44) $$\begin{array}{c} \tilde{x}(k+1)=\left[\begin{array}{rrr} 0.1454 & -0.0418 & 0.0447\\ 0.7084 & 0.1740 & 0.1958\\ 0.0246 & 0.0061 & -0.0620 \end{array}\right]\;\tilde{x}(k)+[\begin{array}{rr} 0.0813 & 0.0813\\ 0.2741 & 0.2741\\ -0.0049 & -0.0049 \end{array}]\;y_{c}^{{ℱ}_{i}}(k)\\ \tilde{z}(k)=\left[\begin{array}{rrr} 0.7055 & 0.8941 & -0.0583\\ -1.2318 & -0.7672 & 1.4227 \end{array}\right]\;\tilde{x}(k) \end{array}$$

**Figure 3.**
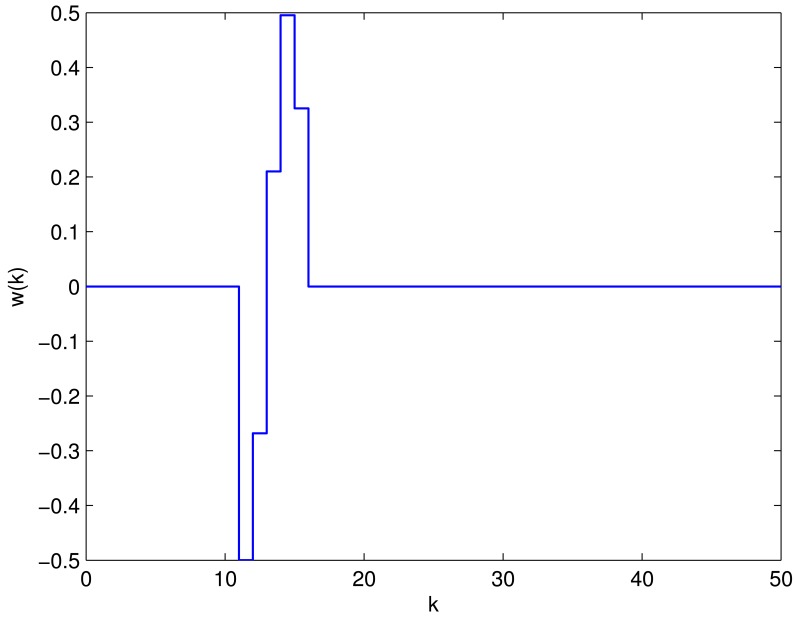
The disturbance input *w*(*k*).

In the following, the system is simulated in low frequency domain, where the faults always occur, under the following two fault modes with zero initial condition and the disturbance input *w*(*k*) is

$$w(k)=\{\begin{array}{ll} 0.5\sin(k), & 10\leq k\leq 15\\ 0, & \text{otherwise} \end{array}$$

which is shown in Figure 3.

### Mode 1

The first sensor being stuck at 0, *i.e.*,

$$F=\left[\begin{array}{ll} 0 & 0\\ 0 & 1 \end{array}\right]$$

and *y_si_*(*k*) = 0 when *k* > 30.

### Mode 2

The second sensor being stuck at 0, *i.e.*,

$$F=\left[\begin{array}{ll} 1 & 0\\ 0 & 0 \end{array}\right]$$

and *y_si_*(*k*) = 0 when *k* > 30.

**Figure 4.**
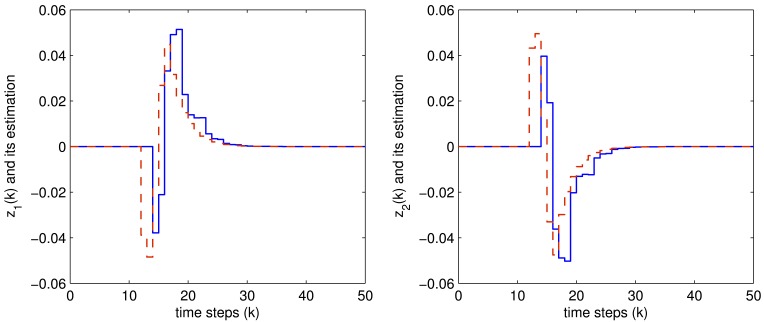
The *z*(*k*) (blue solid) and *z̃*(*k*) (red dashed) when faulty mode 1 occurred.

**Figure 5.**
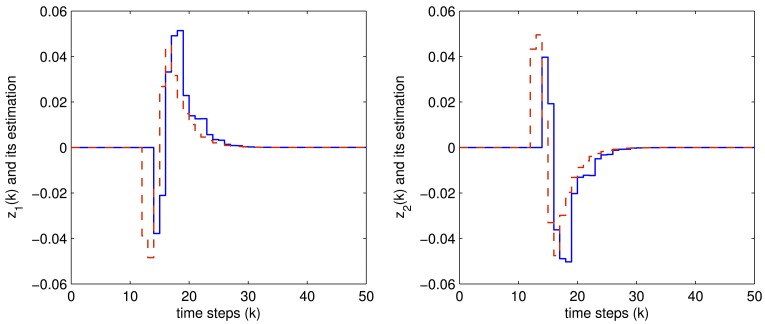
The *z*(*k*) (blue solid) and *z̃*(*k*) (red dashed) when faulty mode 2 occurred.

The controlled outputs and the corresponding estimations for both the two fault modes are shown in Figures 4 and 5, respectively, where the blue solid lines are the controlled output while the red dashed lines are their estimations.

It is easily seen from these figures that all the expected system performance requirement are well achieved, which shows the effectiveness of the proposed method.

## 5. Conclusions

In this paper, the reliable finite frequency filtering problem for NCSs subject to quantization and packet have been studied with finite frequency specifications when sensor fault would occur. The considered NCSs have been first modeled as a discrete time-delay switched system. Subsequently, a sufficient condition to characterize the finite frequency *l*_2_ gain has been presented. Then by virtues of the derived condition, a procedure of reliable filter synthesis has been derived in terms of LMIs. Finally, an example has been given to illustrated the effectiveness of the proposed method.
